# Relational Harm Reduction for Internists: A Call to Action

**DOI:** 10.1007/s11606-024-08693-y

**Published:** 2024-02-29

**Authors:** Mary Hawk, Emma Sophia Kay, Raagini Jawa

**Affiliations:** 1 Behavioral and Community Health Sciences, University of Pittsburgh School of Public Health, 6127 Public Health, 130 DeSoto Street, Pittsburgh, PA 15261 USA; 2 School of Nursing, University of Alabama at Birmingham, Birmingham, AL USA; 3 Center for Research On Healthcare, Division of General Internal Medicine, Department of Medicine, University of Pittsburgh School of Medicine, Pittsburgh, PA USA

## The Opioid Crisis

Drug overdose deaths continue to rise in the United States with over 106,000 fatal overdoses in 2021, primarily related to the toxic drug supply that has replaced heroin with highly potent synthetic fentanyl along with novel adulterants such as xylazine. Overdose affects all ages and geographic locations, and disproportionately impacts racial-ethnic minorities. The per capita overdose mortality rate among non-Hispanic Black individuals more than tripled between 2010 and 2019, in contrast to a 58% rise among non-Hispanic Whites within the same timeframe.^1^ Overdose can occur across the entire spectrum of intermittent use to chaotic use to substance use disorder, truly making it a pervasive epidemic. Federal measures to address this crisis (e.g., elimination of the Drug Enforcement Administration X- Waiver; approval of intranasal naloxone for over-the-counter non-prescription use; designation of xylazine as an emerging threat) have been only sporadically implemented across states and are therefore limited in impact. Racial disparities persist in access to care; Black versus White patients have significantly lower odds of receiving buprenorphine prescriptions during ambulatory visits.^2^ Overall, responses to substance use and substance use disorders are highly racialized. While there are no significant differences in illicit drug use by race, policies around treatment, criminalization, and incarceration have much more deleterious effects on Black people compared to White people.^3^ Further, inadequate access to addiction medicine specialty care, geographic heterogeneity of low-barrier treatment opportunities, and legislative barriers to syringe service programs, all compounded by substance use stigma, contribute to the pervasiveness of this crisis.

## The Shared Role of Internists

Patients are too often expected to shoulder the responsibility of overdose prevention, but it is the duty of the clinical provider to capitalize on every healthcare touchpoint to mitigate overdose and other drug-related harms with the full spectrum of harm reduction care. The potential impact of internists is significant: one study modeling the integration of buprenorphine and harm reduction kits into primary care settings suggests these actions can avert mortality, including overdose, by more than 30%.^4^ Historically, internists have conceptualized harm reduction mainly as the structural services for people who use drugs, such as syringe service programs, prescribing medications for opioid use disorder, and naloxone distribution, i.e., structural harm reduction. However, these strategies vary with local policy, which disadvantages regions with more punitive and regressive attitudes towards drug use. Internists can complement these structural harm reduction strategies with a relational harm reduction approach. Relational harm reduction is grounded in the patient-provider relationship and is defined by the principles of humanism, pragmatism, individualism, autonomy, incrementalism, and accountability. It is a powerful communication tool for all clinical providers that incorporates patient-centric, non-stigmatizing care and promotes patient autonomy.^5^ Relational harm reduction may improve health outcomes even in the absence of supportive policy. This approach may be especially significant for racialized communities since US healthcare and criminal systems have historically caused disproportionate harm to people of color.^3^ Centering patients’ realities calls for internists to acknowledge racial disparities in access to resources, experiences of safety in healthcare settings, and as a result, readiness for open communication about substance use.

Incorporating structural and relational harm reduction in tandem acknowledges the realities of patients who live in communities and states with insufficient access to harm reduction resources. *By leveraging both structural and relational harm reduction strategies, internists have multiple opportunities to mitigate risk of overdose for their patients*. Potential areas for the application of relational harm reduction principles include universal overdose prevention, drug supply, drug preparation, and drug use (Table 1). To demonstrate the potential for relational harm reduction to improve patient care and reduce overdose likelihood, we offer an example of a patient-provider encounter in which structural harm reduction services are limited.

### The Scenario

A 30-year-old male recently diagnosed with severe opioid use disorder comes to your outpatient clinic after an Emergency Room visit for unintentional overdose. He initially experimented with opioid pills 3 years ago, and then progressed to snorting then injecting fentanyl 6 months ago. He has not disclosed his use to his loved ones. He is uninsured and lives in a rural area where syringe service programs are not legal and therefore has resorted to reusing his injection supplies. He has been trying to access buprenorphine treatment and was referred to an addiction services provider an hour away but had trouble finding transportation, experienced long wait times for an intake visit, and was ultimately unable to establish care. He discloses that he has never felt “comfortable” in medical settings due to concerns about being stigmatized for his substance use.

### The Response

Despite limited access to structural resources in your region, there are still many things you as an internist can do to reduce risk and improve the patient’s engagement in care. Understanding he has likely experienced substance use stigma and potentially racial discrimination in his personal life as well as in medical settings, you begin by seeking permission to discuss his substance use, then normalize overdose risk. You explore ways to mitigate injection risks and ask him to show you how and where he injects drugs to assess if there are any injection site skin infections, explore his interest in alternate route of administration which may reduce overdose risk, and prescribe syringes to mitigate equipment reuse.^6^ You also discuss the importance of carrying naloxone, give him a naloxone kit to take home, and help tailor an overdose prevention plan given he typically uses by himself. You suggest he download the free Brave app on his phone, which will enact his overdose response plan if he becomes unresponsive while using.^7^ Although fentanyl testing strips are not legal in his state, you discuss risk mitigation strategies like “test shots” (i.e., first injecting a small amount of the drug to gauge drug potency) and avoiding mixing substances. Lastly, if the patient is still interested in medications for opioid use disorder, you review the availability of a low-barrier buprenorphine induction in the clinic.

## Conclusion

*Every conversation with a patient is an opportunity for internists to save lives*. By tailoring both structural and relational harm reduction approaches, the provider can mitigate the patient’s risk of overdose and drug-related harms especially when structural resources are unevenly distributed across states or difficult for patients to access due to substance use stigma. Harm reduction conversations are particularly important for racialized and other historically oppressed patients who experience disproportionate rates of overdose. Relational harm reduction, which offers patient education, overdose prevention resources, and clinical care in a non-stigmatizing manner, is free and available to all and therefore should be a central component of patient-provider communication. We urge all internists to consider how they might save lives by incorporating these approaches and encourage experienced internists to build these principles into medical education and faculty development programs to mentor and support others in harm reduction care, which also helps to reduce stigma and normalize the diversification of internists working in this space. Still, this work cannot occur in isolation. It will require a paradigm shift across the healthcare system, including changes in reimbursement processes such as incentivizing providers’ time spent communicating with patients about harm reduction. Above all, it will require a culture change and ownership of structural and relational harm reduction by internists.

**Table 1:** Internists’ Overdose Toolkit for Structural and Relational Harm Reduction

Intervention focus	Structural harm reduction	Relational harm reduction
Universal precautions	● Opportunities to enhance naloxone access ○ Open prescription ○ Prescriptions with refills ○ Over-the-counter access ○ Onsite point of care ○ Vending machines ○ Free local access points ○ Good Samaritan laws ● Safe storage and disposal of drug use equipment ● Prescription Drug Monitoring Program monitoring	● Normalizing conversations about substance use with all patients rather than waiting for patients to disclose substance use concerns ● Prioritize patients’ goals; do not assume abstinence as the only positive outcome ● Establish policies ensuring patients can continue in care even in the presence of ongoing substance misuse ● Normalizing conversations about toxic drug supply and risk of overdose ● Discuss concepts of opioid tolerance and relapse ● Dispel myths and stigma around fentanyl, overdose, and naloxone ● Ask about prior overdoses or knowledge of signs and symptoms of overdose and overdose antagonists ● Counsel on signs and symptoms of overdose and how to use intranasal naloxone in clinic and/or at discharge ● Ask about barriers to access including copayment, transportation to care, and stigma ● Tailor overdose response plans ● Have open conversations about drug use, pain, mental health, concurrent substance use disorder, stigma, and self- treatment with substances ● Provide regular and inclusive anti-stigma trainings for healthcare workers
Drug supply	● Distribute onsite fentanyl and/or xylazine testing strips in areas where these are legal	● Know low barrier drug checking access points ● Develop partnerships with drug checking programs and harm reduction organizations to know about local supply ● Advocate for safe supply and provide access to opioid agonists
Drug preparation	● Distribute harm reduction kits on site ● Prescribe sterile syringes	● Discuss alternate routes of use (e.g., switching from intravenous route to oral or intranasal route) ● Review patient’s overdose prevention plan (e.g., never using alone, taking turns, starting low and going slow when using, having naloxone on hand, avoiding mixing substances) ● Refer to safe consumption site/overdose prevention centers or sobering centers
Drug use	● Lower barrier medication for opioid use disorder (MOUD) ○ Linkage with methadone maintenance therapy programs ○ Offering rapid inductions ○ Non-punitive urine drug screens ○ Regular, non-stigmatizing assessment for interval use, cravings, etc	● Virtual or phone-based overdose prevention platform (e.g., neverusealone.com, Brave application) ● Check drug potency: “test shot”
